# Cbl-b-regulated extracellular signal-regulated kinase signaling is involved in the shikonin-induced apoptosis of lung cancer cells *in vitro*

**DOI:** 10.3892/etm.2015.2283

**Published:** 2015-02-10

**Authors:** DAN QU, YU CHEN, XIAO-MAN XU, MENG ZHANG, YI ZHANG, SHENG-QI LI

**Affiliations:** 1 Department of Respiratory Medicine, Shengjing Hospital of China Medical University, Shenyang, Liaoning 110004, P.R. China; 2 Department of Geriatrics, Shengjing Hospital of China Medical University, Shenyang, Liaoning 110004, P.R. China

**Keywords:** phosphoinositide 3-kinase/Akt, shikonin, apoptosis, Cbl-b, extracellular signal-regulated kinase

## Abstract

Shikonin (SK), a naturally occurring naphthoquinone, exhibits antitumor activity. However, its precise mechanisms of action are unknown. In the present study, the effects of SK on NCI-H460 human lung cancer cells were investigated. It was found that SK reduced cell viability and induced apoptosis in the NCI-H460 cells. Additionally, SK inhibited extracellular signal-regulated kinase (ERK) activity, which indicates that inhibition of the ERK pathway is probably one of the mechanisms by which SK induced NCI-H460 cell apoptosis. The expression of Cbl-b was significantly increased by treatment with SK for 4 h, and gradually increased to a maximal level at 24 h; the time taken for the upregulation of Cbl-b protein was in accordance to that required for the downregulation of phospho (p)-ERK protein. The Cbl inhibitor Ps341 reversed the SK-induced downregulation of p-ERK and apoptosis of NCI-H460 cells. These results indicate that Cbl-b potentiates the apoptotic action of SK by inhibiting the ERK pathway in lung cancer cells.

## Introduction

Lung cancer is the leading cause of cancer mortality worldwide. It accounts for 22% of total cancer mortalities with only a 7% 5-year survival expectancy in the United Kingdom (1). Non-small cell lung cancer (NSCLC) accounts for ~80% of all diagnosed cases of lung cancer (2). Chemotherapy plays an important role in the treatment of NSCLC. Anticancer drugs exert their therapeutic action mainly by inducing tumor cell apoptosis. However, the non-selective killing and toxicity of traditional chemotherapy drugs limit their clinical application. In recent years, Chinese herbal medicines have attracted attention for use as anti-neoplastic medicines and adjuvant chemotherapeutic agents. Accordingly, new therapeutic methods are worthy of development.

**Figure 1:**
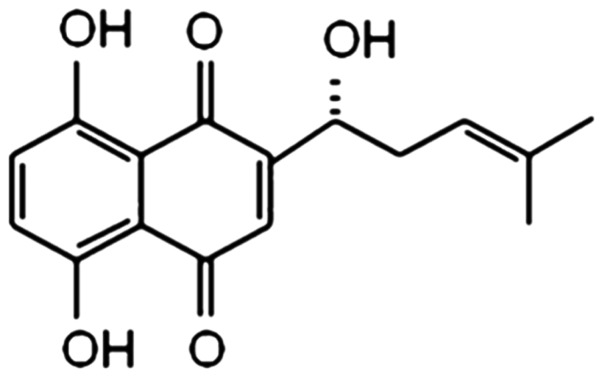
Chemical structure of shikonin.

Shikonin (SK) is the major active ingredient isolated from the dried roots of *Lithospermum erythrorhizon*, with a molecular weight of 288 (Fig. 1). As a naphthoquinone pigment, SK exhibits multiple biological activities such as anti-inflammatory, antimicrobial and antitumor effects, as well as antagonism of the human immunodeficiency virus (HIV) and the promotion of wound-healing (3–5). Previous studies have demonstrated that SK can induce apoptosis in various tumor cells, suggesting that it could provide a novel option for antineoplastic therapy (6,7).

Cell survival depends on the balance of apoptosis and proliferation signals. The phosphoinositide 3-kinase (PI3K)/Akt and extracellular signal-regulated kinase (ERK) signaling pathways are the two major pro-proliferative and anti-apoptotic pathways; by affecting the activation of downstream apoptosis-related proteins and cell cycle regulatory proteins, they play important roles in tumor cell proliferation, angiogenesis and metastasis as well as in antagonistic chemotherapy. Studies have shown that components of the PI3K/Akt and ERK signaling pathways are usually overexpressed or activated excessively in numerous types of cancer, including gastric, colon, lung and breast cancer (8–10). Inhibition of the two pathways may increase the sensitivity of tumor cells to cytotoxic drugs. However, whether SK induces apoptosis in NCI-H460 lung cancer cells by affecting the PI3K/Akt and/or ERK pathways is not known.

Cbl-b is one of the key members of the Cbl family of ubiquitin ligases; it can induce the ubiquitination and degradation of a variety of proteins, and participate in receptor tyrosine kinase signaling (11). A number of studies have found that Cbl-b can negatively regulate the PI3K/Akt and ERK pathways by the ubiquitination of PI3K and ERK (12–14). However, it remains unclear whether Cbl-b is involved in SK-induced apoptosis. In the present study, whether Cbl-b potentiates the apoptotic action of SK by inhibiting the ERK and/or PI3K/Akt pathways was investigated in human NCI-H460 large-cell lung carcinoma cells.

## Materials and methods

### Reagents and antibodies

Mouse anti-human monoclonal anti-phospho (p)-ERK (cat. no. sc-7383) and rabbit anti-human polyclonal anti-Cbl-b (cat. no. sc-1704) antibodies were purchased from Santa Cruz Biotechnology (Santa Cruz, CA, USA). Rabbit anti-human monoclonal anti-p-Akt (Ser-473) (cat. no. 3787s), rabbit anti-human polyclonal anti-Akt (cat. no. 9272) and rabbit anti-human polyclonal anti-ERK1/2 (cat. no. 9102s) antibodies were purchased from Cell Signaling Technology (Beverly, MA, USA). Rabbit anti-human polyclonal anti-β-actin antibodies (cat. no. ab16039) were purchased from Abcam (Cambridge, UK). SK (>98% pure) was purchased from the National Institute for the Control of Pharmaceutical and Biological Products (Beijing, China). SK was dissolved in dimethyl sulfoxide (DMSO) at a stock concentration of 100 mM, aliquoted, and stored at −20°C. Fetal bovine serum (FBS) was purchased from Solarbio Science & Technology Co., Ltd. (Beijing, China). 3-(4,5-Dimethyl-thiazol-2-yl)-2,5-diphenyltetrazolium bromide (MTT), DMSO and Hoechst 33342 were purchased from Sigma-Aldrich (St. Louis, MO, USA). The annexin V-fluorescein isothiocyanate (FITC) and propidium iodide (PI) double staining kit was purchased from Biosea Biotechnology Co., Ltd. (Beijing, China).

### Cell culture

The NCI-H460 human large-cell lung carcinoma cells were purchased from the Department of Cell Biology, China Medical University (Shenyang, China). NCI-H460 cells were cultured in RPMI-1640 medium Hyclone; Thermo Fisher Scientific, Rockford, IL, USA) containing 10% FBS, 100 U/ml penicillin and 100 μg/ml streptomycin at 37°C under an atmosphere of 95% air and 5% CO_2_. Cells were routinely subcultured every 2–3 days and the cell samples used were all in the logarithmic growth phase.

### Cell viability assay

Cells were seeded at 0.8×10^4^ cells/well in 96-well plates and incubated overnight. Different concentrations (0.312–10 μM) of SK were then added and the cells were further incubated for 24 and 48 h. Thereafter, 20 μl MTT solution (5 mg/ml) was added to each well and the cells were incubated for another 4 h at 37°C. Following the removal of culture medium, the cells were lysed in 150 μl DMSO and the optical density (OD) was measured at 570 nm using a microplate reader (Model 550; Bio-Rad, Hercules, CA, USA).

### Analysis of cell apoptosis

NCI-H460 cells were plated at a density of 0.8×10^6^ cells per well in 6-well plates overnight and then treated with 2.1 or 2.6 μM SK for 24 h. The cells were then collected. In addition, NCI-H460 cells were plated in 6-well plates (0.8×10^6^cells/well) overnight and were pretreated with 10 nM Ps341 (Active Biochem, Maplewood, NJ, USA), an inhibitor of proteasome and Cbl, for 1 h. A total of 2.6 μM SK was then added to the cells for a further 24 h, and the cells were collected. All of the cells were then washed twice with PBS, and incubated with 10 μl annexin V and 5 μl PI for 15 min in the dark. Samples were then evaluated using a FACScan flow cytometer (Becton Dickinson, San Jose, CA, USA).

### Fluorescence microscopy

NCI-H460 cells were treated with 2.1 or 2.6 μM of SK for 24 h. Then, the cells were collected, washed twice with PBS, and fixed in a mixture of cold methanol and acetic acid (3/1, v/v) prior to staining with Hoechst 33342 (1 mg/ml) for 30 min at 37°C. Stained cells were observed with a fluorescence microscope (magnification, ×400; Eclipse 90i, Nikon, Tokyo, Japan).

### Western blot analysis

The expression levels of cellular proteins were evaluated by western blotting. Following treatment with 2.6 μM SK for 24 h, and pretreatment with 10 nM Ps341 or not for 1 h, the cells were washed twice with ice-cold PBS and total proteins were solubilized and extracted with lysis buffer (20 mM HEPES pH 7.9, 20% glycerol, 200 mM KCl, 0.5 mM EDTA, 0.5% NP-40, 0.5 mM DTT and 1% protease inhibitor cocktail). Protein concentration was determined by the bicinchoninic acid (BCA) protein assay. Equal amounts of protein (50 μg) from each sample were separated by SDS-PAGE. Following electrophoresis, proteins were electroblotted to polyvinylidene difluoride membranes. The membranes were blocked with 5% nonfat dry milk at room temperature for 1 h and then independently incubated at 4°C overnight with the primary antibodies against ERK (1:1,000), p-ERK (1:1,000), Akt (1:1,000), p-Akt (1:1,000), or Cbl-b (1:250), and β-actin (1:1,000) was used as a control. After washing three times with TBST (20 mM Tris-Cl pH 7.5, 150 mM NaCl and 1 g/l Tween 20), the membranes were incubated with horseradish peroxidase-conjugated goat anti-mouse (cat. no. A0216) and goat anti-rabbit (cat. no. A0208) secondary antibodies (1:800 dilutions; Beyotime Institute of Biotechnology, Shanghai, China), followed by a further three washes with TBST. Proteins bands were visualized using an enhanced chemiluminescent reagent (Thermo Fisher Scientific).

### Statistical analysis

The data are expressed as the mean ± standard deviation. Statistical correlation of data was checked for significance by two-way analysis of variance and Student’s t-test, using SPSS version 17.0 software (SPSS Inc., Chicago, IL, USA). P<0.05 was considered to indicate a statistically significant difference.

## Results

### SK inhibits NCI-H460 cell growth

**Figure 2:**
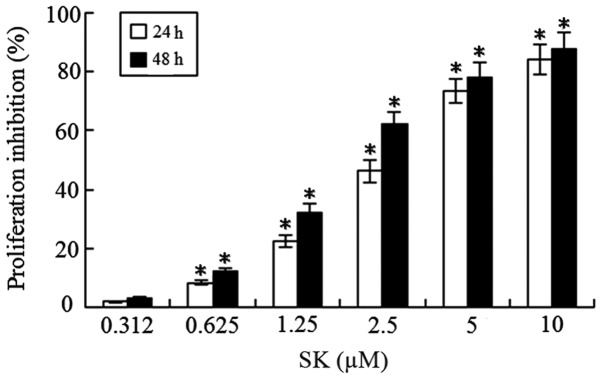
Proliferation-inhibiting effects of shikonin (SK) on NCI-H460 human lung cancer cells. Logarithmically growing NCI-H460 cells were treated with the indicated concentrations of SK for either 24 or 48 h. Cell viability was then assessed by 3-(4,5-dimethylthiazol-2-yl)-2,5-diphenyltetrazolium bromide assay. ^*^P<0.01 compared with the control (0 μM SK) group.

To determine whether SK inhibits the proliferation of lung cancer cells, NCI-H460 cells were treated with different concentrations of SK (0.312–10 μM) for 24 or 48 h. A significant concentration- and time-dependent reduction of cell viability was observed (Fig. 2). The 50% inhibitory concentration (IC_50_) of SK for the NCI-H460 cells at 24 and 48 h was 2.64±0.52 and 1.75±0.28 μM, respectively.

### SK induces NCI-H460 cell apoptosis

**Figure 3:**
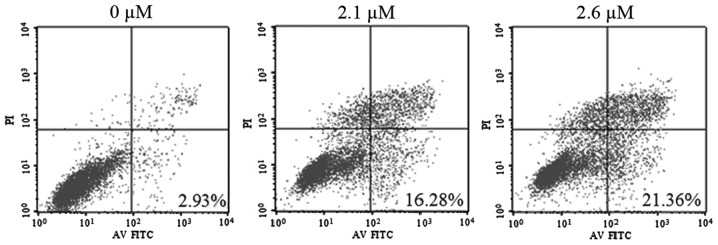
Apoptosis analysis by flow cytometry. NCI-H460 cells were treated with the indicated concentrations of shikonin for 24 h. Changes in cell apoptosis distribution were assessed by annexin V fluorescein isothiocyanate (AV FITC)/propidium iodide (PI) staining and flow cytometry. The percentages of apoptotic (double-stained) cells are indicated.

**Figure 4:**
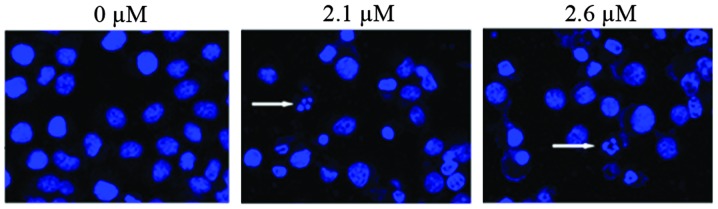
Apoptosis analysis by fluorescence microscopy. NCI-H460 cells were treated with the indicated concentrations of shikonin for 24 h, then apoptosis was observed by Hoechst 33342 staining and analysis by confocal microscopy. Chromatin condensation and nuclear fragmentation are indicated by the white arrowheads. Magnification, ×400.

To determine whether SK induces apoptosis in NCI-H460 cells, SK-induced cytotoxicity was examined. Flow cytometric analysis with Annexin V and PI demonstrated that SK induced significant apoptosis in NCI-H460 cells. The percentages of double-stained cells, indicative of apoptosis, were 16.28±2.18% (P<0.01) and 21.36±2.67% (P<0.01) following treatment with 2.1 and 2.6 μM SK, respectively, compared with 2.93±0.23% in the non-treated control (Fig. 3). Consistent with this, confocal fluorescence microscopy revealed clear morphological changes typical of apoptosis, such as condensation of chromatin and nuclear fragmentation, following treatment with SK (Fig. 4).

### SK induces NCI-H460 cell apoptosis by inhibiting ERK signaling

**Figure 5:**
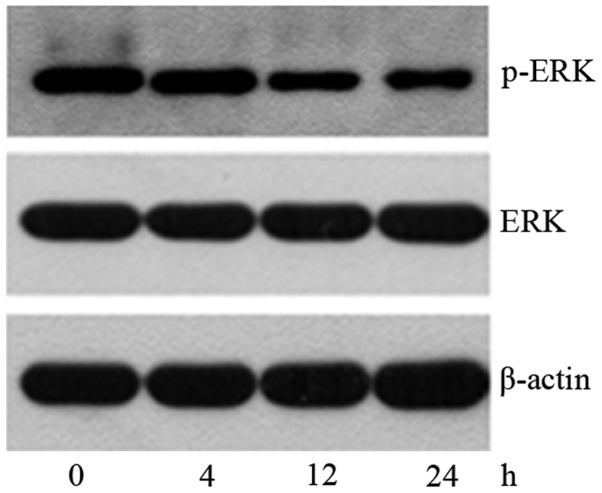
Effects of shikonin (SK) on the extracellular signal-regulated kinase (ERK) signaling pathway in NCI-H460 cells. NCI-H460 cells were treated with 2.6 μM SK for the indicated times. The expression levels of phospho (p)-ERK and ERK proteins were then analyzed by western blotting.

**Figure 6:**
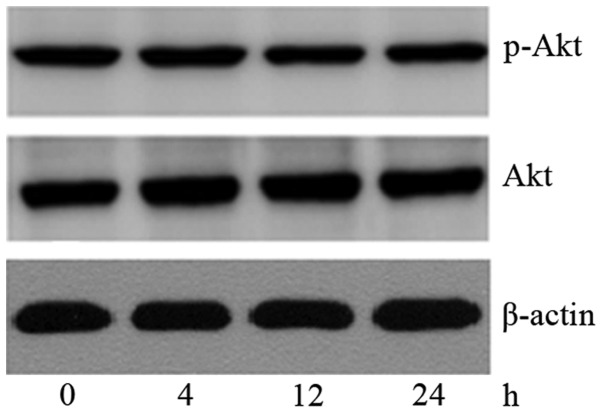
Effects of shikonin (SK) on the phosphoinositide 3-kinase (PI3K)/Akt signaling pathway in NCI-H460 cells. NCI-H460 cells were treated with 2.6 μM SK for the indicated times. The expression levels of phospho (p)-Akt and Akt proteins were then analyzed by western blotting.

To further determine the mechanism of SK-induced apoptosis in NCI-H460 cells, the levels of p-Akt, total Akt, p-ERK and total ERK were investigated following treatment with 2.6 μM SK for 4–24 h. It was found that the expression of p-ERK protein began to decrease at 4 h, and reached a minimum expression level at 24 h (Fig. 5), while the expression level of p-Akt was not changed (Fig. 6). This indicates that SK induced NCI-H460 cell apoptosis by inhibiting the ERK signaling pathway but not the PI3K/Akt pathway.

### Cbl-b contributes to the SK-induced apoptosis of NCI-H460 cells by negatively regulating ERK signaling

**Figure 7:**
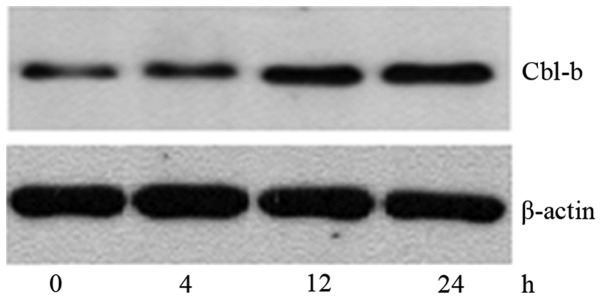
Effect of shikonin (SK) on the expression of Cbl-b protein in NCI-H460 cells. NCI-H460 cells were treated with 2.6 μM SK for 4–24 h, as indicated. The changes in the protein expression levels of Cbl-b were assessed by western blotting.

To explore whether the inactivation of ERK is associated with Cbl-b, the protein expression level of Cbl-b was evaluated by western blotting (Fig. 7). The expression of Cbl-b was significantly increased by treatment with SK for 4 h, and gradually increased to a maximal level at 24 h; the time taken for the upregulation of Cbl-b protein was in accordance with that required for the downregulation of p-ERK protein. This indicates that the inactivation of ERK is probably associated with the upregulation of Cbl-b. The results indicate that there is a correlation between Cbl-b and p-ERK. To confirm this hypothesis, NCI-H460 cells were pretreated with Ps341, a proteasome and Cbl inhibitor for 1 h; then, 2.6 μM SK was added for a further 24 h. It was found that Ps341 reversed the SK-induced downregulation of p-ERK and apoptosis of NCI-H460 cells (Fig. 6). These data suggest that Cbl-b potentiates the apoptotic action of SK by inhibiting the ERK pathway in lung cancer cells.

## Discussion

SK, one of the major naphthoquinone pigments isolated from the traditional Chinese herb *Lithospermum erythrorhizon*, has numerous biological functions. Recent studies have shown that SK can induce apoptosis in liver cancer, osteosarcoma and prostate cancer cells (15–17). SK exerts antitumor effects by upregulating the expression of p53 protein, regulating the expression of Bcl-2 family proteins, inducing reactive oxygen species (ROS) production, promoting ERK and c-Jun N-terminal kinase (JNK) phosphorylation, inhibiting epidermal growth factor receptor (EGFR) and protein tyrosine kinase (PTK) phosphorylation and the activity of topoisomerase, telomerase and matrix metalloproteinase-9 (MMP) (18–20). In the present study, it was confirmed that SK inhibits cell proliferation in a time- and concentration-dependent manner and induces the apoptosis of NCI-H460 cells.

The PI3K/Akt pathway plays a key role in maintaining cell survival and inhibiting apoptosis. Activated Akt exerts anti-apoptotic activity by phosphorylating Bad and caspase 9 and activating the transcription factor NF-kB (21). Numerous cytotoxic drugs induce cell apoptosis by inhibiting the PI3K/Akt pathway. ERK is associated with the mitogen-activated protein kinase (MAPK) pathway; it plays an important role in tumor incidence and development by inducing tumor cell differentiation and proliferation. Indeed, PI3K/Akt and ERK signaling pathways are excessively activated in some cancer cells, while their inhibition can increase the sensitivity of tumor cells to cytotoxic drugs (22–24). SK has been shown to increase ROS generation and ERK activation, and induce apoptosis in osteosarcoma cells (16). Phosphorylated ERK has been shown to upregulate p53 expression in SK-induced HeLa cell apoptosis (20). In the present study, the treatment of cells with 2.6 μM SK for 4–24 h resulted in the gradually decreased expression of p-ERK protein, which reached a minimum at 24 h, while the expression of p-Akt did not change. Typical apoptosis was observed at 24 h, suggesting that SK first inhibited the ERK pathway, which in turn, blocked its regulation of downstream factors, resulting in the loss of ERK pathway-mediated anti-apoptotic function the and induction of NCI-H460 cell apoptosis. This indicates that inhibition of the ERK pathway is probably one of the mechanisms by which SK induces the apoptosis of NCI-H460 cells.

The Cbl family of ubiquitin ligases comprises adaptor proteins and E3 ligases that play both positive and negative roles in several signaling pathways and affect various cellular functions (25–27). The results of the present study showed that the expression of Cbl-b began to increase when the NCI-H460 cells had been exposed to 2.6 μM SK for 4 h, and reached maximal expression at 24 h. The kinetics of the upregulation of Cbl-b corresponded with those of ERK inhibition, suggesting that SK promotes ERK ubiquitination by upregulating the expression of Cbl-b, which in turn inhibited ERK activity and finally induced NCI-H460 cell apoptosis. Ps341, a Cbl inhibitor, reversed both SK-induced cell apoptosis and inhibition of p-ERK activity. These findings confirm that Cbl-b is involved in SK-induced NCI-H460 cell apoptosis by negatively regulating ERK activity.

In summary, the results of the present study indicate that SK induces apoptosis in NCI-H460 cells and inhibits their proliferation. Cbl-b-regulated ERK signals are involved in SK-induced cell apoptosis *in vitro*. This study indicates that SK may serve as an important potential chemotheraputic agent in human lung cancer.

## Figures and Tables

**Figure 8:**
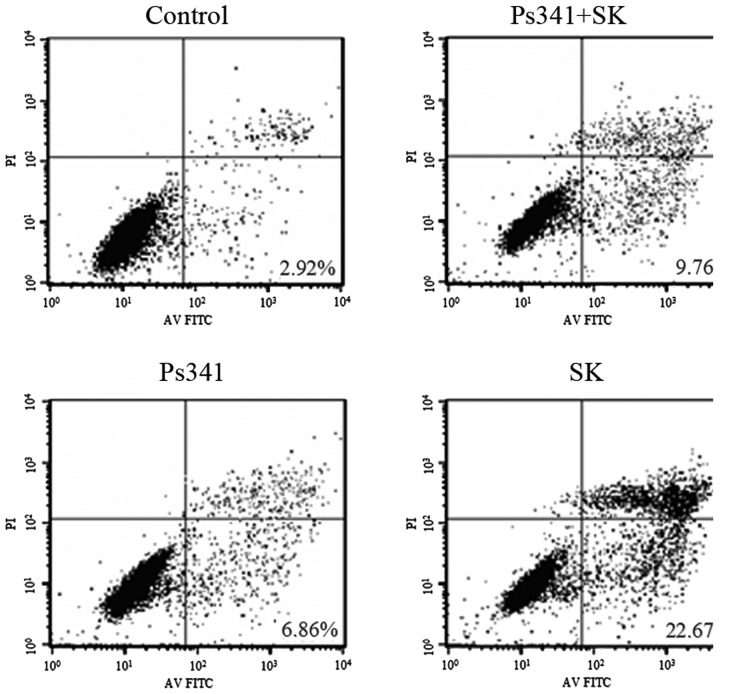
Effects of the combined application of Ps341 and shikonin (SK) on apoptosis in NCI-H460 cells. NCI-H460 cells were pretreated with the Cbl inhibitor Ps341 for 1 h, then 2.6 μM SK was added for a further 24 h. The changes in cell apoptosis distribution were assessed by annexin-V fluorescein isothiocyanate (AV FITC)/propidium iodide (PI) staining followed by flow cytometric analysis. The percentages of apoptotic cells are indicated.

**Figure 9:**
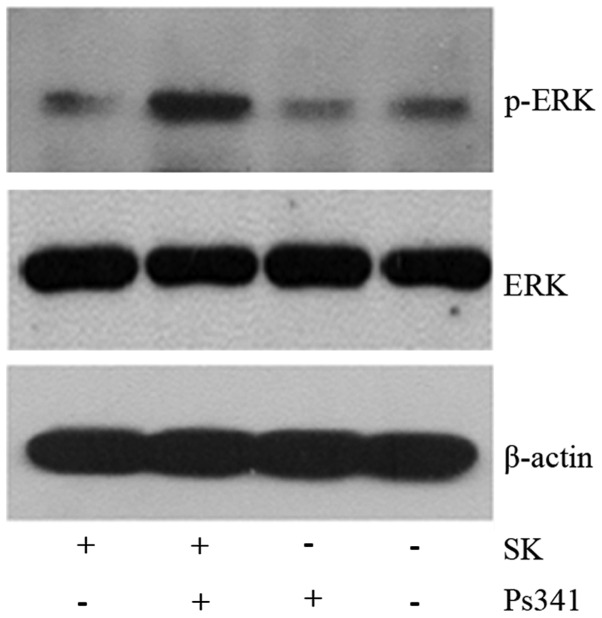
Effects of the combined application of Ps341 and shikonin (SK) on the expression of phospho (p)-extracellular signal-related kinase (ERK) and ERK proteins in NCI-H460 cells. NCI-H460 cells were pretreated with the Cbl inhibitor Ps341 for 1 h, then 2.6 μM SK was added for a further 24 h. The protein expression levels of p-ERK, ERK and β-actin were analyzed by western blotting.
